# Optimized Design of Neural Networks for a River Water Level Prediction System

**DOI:** 10.3390/s21196504

**Published:** 2021-09-29

**Authors:** Miriam López Lineros, Antonio Madueño Luna, Pedro M. Ferreira, Antonio E. Ruano

**Affiliations:** 1 Design Engineering Department, University of Seville, 41013 Seville, Spain; 2 Aerospace Engineering and Fluid Mechanical Department, University of Seville, 41013 Seville, Spain; amadueno@us.es; 3 LASIGE, Departamento de Informática, Faculdade de Ciências, Universidade de Lisboa, 1749-016 Lisboa, Portugal; pmf@ciencias.ulisboa.pt; 4 Faculty of Science and Technology, University of Algarve, 8005-139 Faro, Portugal; aruano@ualg.pt; 5 IDMEC, Instituto Superior Técnico, Universidade de Lisboa, 1049-001 Lisbon, Portugal

**Keywords:** Multi-Objective Genetic Algorithm, artificial neural networks, river stage data

## Abstract

In this paper, a Multi-Objective Genetic Algorithm (MOGA) framework for the design of Artificial Neural Network (ANN) models is used to design 1-step-ahead prediction models of river water levels. The design procedure is a near-automatic method that, given the data at hand, can partition it into datasets and is able to determine a near-optimal model with the right topology and inputs, offering a good performance on unseen data, i.e., data not used for model design. An example using more than 11 years of water level data (593,178 samples) of the Carrión river collected at Villoldo gauge station shows that the MOGA framework can obtain low-complex models with excellent performance on unseen data, achieving an RMSE of 2.5 × 10^−3^, which compares favorably with results obtained by alternative design.

## 1. Introduction

The continuous occupation of the territory by human beings results in a growing population within lowland and riverbanks, which can be affected by floods. The considerable human, economic and social costs resulting of floods forces decision makers and planners to take effective measures to develop territorial policies in order to prevent and manage flood events. These measures involve an exhaustive study and the processing of data collected from telemetric network sensors spread over the river basin in order to understand and predict the hydraulic and hydrological behavior of the river basin [1]. This requires the development of a data assimilation system including exhaustive exploration data from the data acquisition system.

Hydrological/hydraulic models are essential to decision support systems to anticipate and estimate the effects of river floods as well as for water management and drought monitoring.

The prediction of the water level in rivers has been studied by other authors using various techniques, for example: a distributed rainfall-runoff model and a filtering technique [2], Machine Learning Models such as artificial neural network (ANN), decision tree (DT), random forest (RF), support vector machine (SVM) [3], Long Short Term Memory (LSTM) models: a type of neural network ideally suited to predict time-dependent data [4], Artificial Intelligence (AI) and small data sets [5], Artificial Neural Network and Time Series Forecasting Models [6], A Neuro-Fuzzy Approach [7], Support Vector Regression (SVR) [8], Neural Network Autoregressive with Exogenous Input (NN-ARX) [9], deep convolutional neural networks (DeepCNNs) [10], an adaptive neuro-fuzzy inference system [11] or patented models very robust against data failures like [12]. 

There is a lack of a global tool providing data integrity and validated data from real-time information systems so as to input consistently reliable data to hydrologic/hydraulic models, which in turn support relevant decision makers. This paper is focused on the first challenge: the development of intelligent models for predicting the water level in rivers. The availability of these models can afterwards be used to address the second challenge: data validation.

Over the past 25 years, Artificial Neural Networks (ANN) have been increasingly used for prediction and forecasting in water resources. The large number of research activity in this area led to a number of review papers [13,14,15,16,17,18], which not only confirmed the potential of ANNs for the prediction and forecasting of water resource variables but also identified a number of challenges that needed to be addressed in order to ensure that ANNs become a mature modelling approach that can sit comfortably alongside other approaches in the toolkit of hydrological and water resource modelers.

One aspect that needs to be considered in most of the proposals is how to select the inputs of the ANNs. As the ANN models are used to represent the dynamics of the modelled variable, this is translated into selecting the delays to use in the modelled variable and, if existing, in the exogenous variables. Another aspect is how to select the model topology, i.e., the number of neurons and/or the number of hidden neurons of the ANN models. And finally, to have a design procedure that, given the data at hand, can partition it into datasets and is able to determine a near-optimal model, with the right topology and inputs, offering a good performance on unseen data, i.e., data not used for model design.

In this paper, a MOGA design framework is applied to water level modelling of Villoldo gauge station, located in the basin of the river Carrión, an affluent of the Duero River. It provides, as it can be seen in the following sections, a nearly automated way to address all of the challenges identified above. The layout of the paper is as follows: Section 2 described the ANN model used, the MOGA procedure used for model design and the data used for it. Section 3 illustrates the results obtained in two consecutive experiments. Section 4 draws conclusions and points out directions for future research.

## 2. Materials and Methods

### 2.1. Radial Basis Function Neural Networks

Considering the higher model complexity and hardware requirements that DeepCNNs or LSTM networks impose, we opted for a simpler, well-established, type of feed-forward ANN that can easily be implemented in the software framework of most embedded processors used in modern energy efficient cyber-physical systems suitable for deployment in the field. Other techniques such as SVMs or RFs were not considered since, according to our survey, in most regression-based applications they do not extrapolate and generalize as well as ANNs.

Radial Basis Function (RBF) Neural Networks (NN) [19] are fully-connected feed-forward NNs. They are generic function approximators that, provided with sufficient computing units and enough data, can approximate arbitrarily well certain types of functions. RBF NNs consist of three functionally distinct layers: the input layer is simply a set of sensory units; the second layer, also known as hidden layer, performs a non-linear transformation of the data in the input layer; and, finally, the third layer performs a linear combination of its inputs in order to generate the network output. The second layer units, known as neurons, are nonlinear functions of their vector inputs, given by

(1) $$\varphi_{i}\left(x,c_{i},\sigma_{i}\right)=e^{\frac{-\|x-c_{i}\|{}^{2}}{2\sigma_{i}^{2}}},\;\;\;\;\;\;\varphi_{0}=1,$$

where ‖ ‖ denotes the Euclidean norm and *c_i_* and $\sigma_{i}$ are, respectively, the location of the Gaussian function in input space and its spread. Therefore, the RBFNN output is given by

(2) $$\hat{y}\left(x,\alpha ,C,\sigma \right)=\overset{n}{\underset{i=0}{\sum }}\alpha_{i}\varphi_{i}\left(x,c_{i},\sigma_{i}\right)=\varphi \left(x,C,\sigma \right)\alpha ,$$

where *n* is the number of neurons and the *α_i_* are the weights of the network output linear combiner.

Training an RBFNN using a data set D={X,y} corresponds to finding the vector of parameters w=[α,C,σ] that minimises the training criterion:

(3) $$\Omega \left(X,w\right)=\frac{1}{2}\|y-\hat{y}{\left(X,w)\right\|}^{2}.$$

The 1/2 factor is only a scaling factor used for convenience in the formulation of the training method. This least-squares criterion expresses the objective of minimizing the fitting error that the RBFNN achieves when approximating the target output values, y.

It is generally accepted that, for non-linear least-squares, the Levenberg-Marquardt (LM) [20,21] method is the best, as it exploits the sum-of-squares characteristic of the problem [22]. In this work, the LM training method is employed, using a modified training criterion [23] that reflects the non-linear/linear topology of the RBFNN. Furthermore, the modified criterion enables the usage of appropriate methods dedicated to computing each type of the parameters (linear, *α*; and non-linear, *σ* and *c*) in the minimization of a single explicit criterion, it lowers the dimensionality of the problem and it usually achieves an increased convergence rate.

The training is stopped by using the common early stopping method within a sufficiently large number of iterations. Further details about the training methodology, the modified training criterion and the stopping methodology may be found in a more specialized publication [24].

### 2.2. RBF NN Design Using Multi-Objective Genetic Algorithms

The training method briefly described in the preceding section solves the task of fitting the RBF NN parameters to a given data set of input-output patterns. Besides determining the network parameters, designing an RBF NN for a given application involves also determining the appropriate topology (or structure) of the model. In fact, this may be viewed as another optimization problem. The problem has been formulated and analyzed in detail in previous work [24]. In the following subsections, an overview of the approach is presented.

For this work, the design problem consists in the selection of the model inputs (the components of x in Equation (1)) and its number of neurons (*n* in Equation (2)).

#### 2.2.1. Overview

The Multi-Objective Genetic Algorithm (MOGA) [25,26] is an evolutionary algorithm that employs procedures and operators inspired by the natural evolution process and by the notion of survival of the fittest. It performs a population-based search for the Pareto set of solutions to a given problem. Individually, the solution candidates are called individuals, and, collectively, they are termed the population. One run of the MOGA starts with an initial population, the initial generation, which is then evaluated and manipulated to generate the population in the next generation. Hopefully, after enough generations, the population has evolved to an extent that includes a set of individuals that achieve a satisfactory approximation to the Pareto front of solutions.

**Figure 1:**
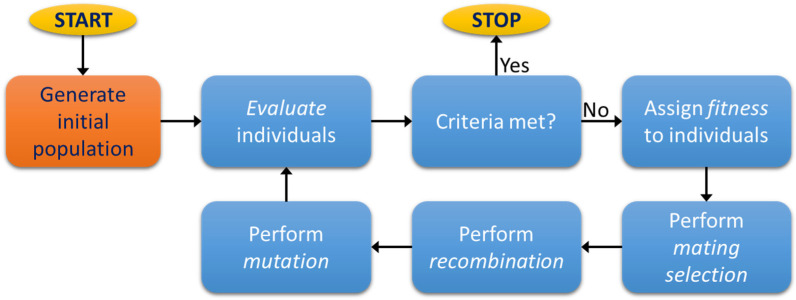
Typical workflow in MOGA operation.

The operation of the MOGA follows the workflow pictured in Figure 1. At each iteration, the candidate solutions are evaluated accordingly to specified objectives and restrictions, and a verification is done to check if the stopping criteria is met. If this is true, the designer receives the individuals that compose the current Pareto set of solutions. Otherwise, the algorithm proceeds by assigning a fitness value to each individual and by mating the individuals according to their fitness. Each pair will then produce two offspring by the application of a recombination operator, hence producing the next generation. Finally, the mutation operator is applied to each new individual before repeating the entire process.

In this paper, we follow the approach developed previously [24], where each individual in the population is specified by a representation that encodes the topology of an RBF NN. This topology is completely specified by the number of neurons, n, and by the specification of each input included in the model. As a simple auto-regressive predictive approach is adopted, from an input-output perspective, the model may be specified by

(4) $${\hat{y}}_{k+1}=f\left(y_{k-\tau_{1}},y_{k-\tau_{2}},\;\ldots ,\;y_{k-\tau_{d}}\right),$$

where $\tau_{1}$ to $\tau_{d}$ specify a set of output delays ranging from 0 to a maximum delay M that are fed back to the model input. Therefore, the chromosome may be represented by an array of integer values where the first element is simply the number of neurons of the model and the $\lambda_{i}$ values are indices to a lookup table of M available input terms, as shown in (4).

#### 2.2.2. Procedures and Operators

The parameters of each individual (each RBF) are estimated according to the method discussed in Section 2.2 After evaluation, a scalar fitness value that reflects that individual’s quality is assigned. Then, as described in previous work [24], the mating procedure uses the fitness information to set up a mating pool with pairs of individuals that will be combined to form the starting point of the next generation. Mating is implemented as a sampling procedure where individuals having higher fitness have increased probability of placing multiple copies in the mating pool and those having lower fitness have little or no chance of reaching the pool. After mating, with a given crossover probability, the recombination operator will exchange part of the chromosome of each individual in a pair to produce two offspring. In this work, the single-point crossover approach was followed.

Finally, mutation is applied independently in the two distinct parts of the chromosome. The number of neurons is mutated with a given probability by adding or subtracting one neuron to the RBF NN hidden layer. Each input term is mutated, also with some probability, by one of three operations: replacement, addition or removal. First, each term is tested. If mutation is to be applied to the term, it is removed or replaced by a different term from the pool. Then, if the chromosome is not full, one new term may be added from those available in the pool.

#### 2.2.3. Model Design Cycle

Globally, the RBF NN structure optimization problem can be viewed as a sequence of actions that should be repeated until certain design goals specified by the model designer are satisfied. The water level model identification task is approached as described in the MOGA based model design framework [24].

In summary, we must define the relevant set of output delays that should be included at the model input and specify the minimum and maximum allowed number of neurons and inputs. These definitions affect the size of the search space. Then, the data set D is partitioned into three subsets of data for model training (D^t^), model generalization testing (D^g^) and model validation (D^v^). The model parameters are determined by the training algorithm using D^t^. D^g^ is used to check the training error criterion at each iteration of the training procedure for a sufficiently large number of iterations. Then, the model parameters can be recalled from the iteration where the error criterion on D^g^ was minimal regardless of the error criterion on D^t^, as in an *early stopping* procedure. As D^t^ and D^g^ are both used within the MOGA execution to optimize the model structure, the validation set, D^v^, is used to validate the results obtained by the individuals in the Pareto front. This step serves the purpose of avoiding any bias that may have occurred towards the sets involved in the optimization.

The objectives considered for the model structure optimization are the Root Mean Square Error (RMSE) obtained with D^t^ and D^g^ and a model complexity indicator to limit the number of inputs and neurons.

When the analysis of results requires the process to be repeated, two major actions can be taken: redefining the input space by adding or removing input alternatives to the pool; and changing the trade-off surface by changing the objectives and restrictions.

### 2.3. Source of Data

**Figure 2:**
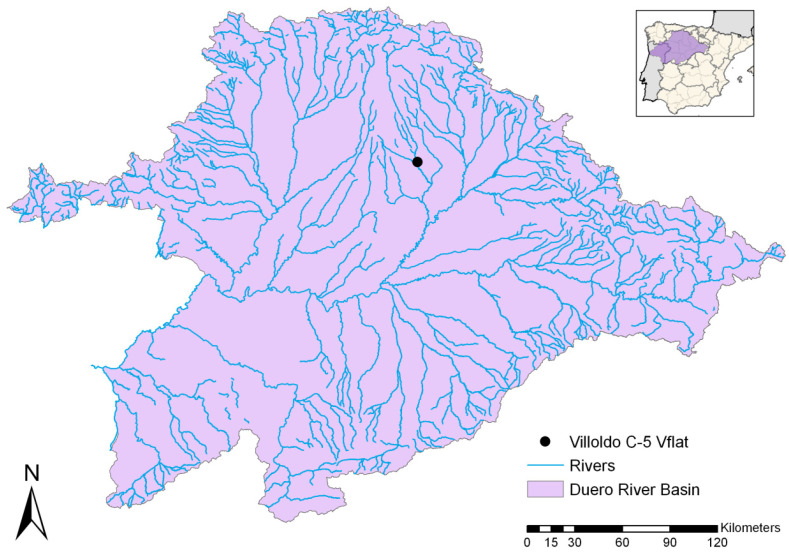
Geographic location of the Duero basin and the Villoldo gauging station.

In this study, river stage data from one gauge station (Villoldo) located in the basin of the River Carrión, an affluent of the Duero River in the northwest of the Iberian Peninsula (Figure 2), have been used.

This station belongs to the Spain National Gauging Network (ROEA), controlled by the Confederación Hidrográfica del Duero (CHD, 2015). It has been selected because it is one of the most reliable gauging points of the Carrión system. Villoldo has been supplying hydrologic information since the first years of the previous century, given the importance of the Carrión River as a water supplier to the Canal of Castile, an artificial canal built between the XVIII and XIX centuries to open a fluvial route to carry the wheat cropped in the area to the ports of the Bay of Biscay. The canal was soon superseded by the railway and was relegated to being a water distribution system for irrigated farms. Currently, the Canal of Castile is a relevant natural park. 

The gauge is a triangular profile V-flat weir, adequate for natural streams (e.g., [27]), converting the water depth into an electrical signal recorded in binary code, (BCD). This signal is sent by cable to a remote station. The remote station adds redundant codes (CRC) for error correcting purposes before re-sending it via satellite to the CPC at 10-min intervals. The BCD decoder can process 20,000 steps with a maximum resolution of 5mm on a 10 m range. At Villoldo gauging station, readings are taken in fractions of 20 steps, which imply a real resolution of 10 mm. 

**Figure 3:**
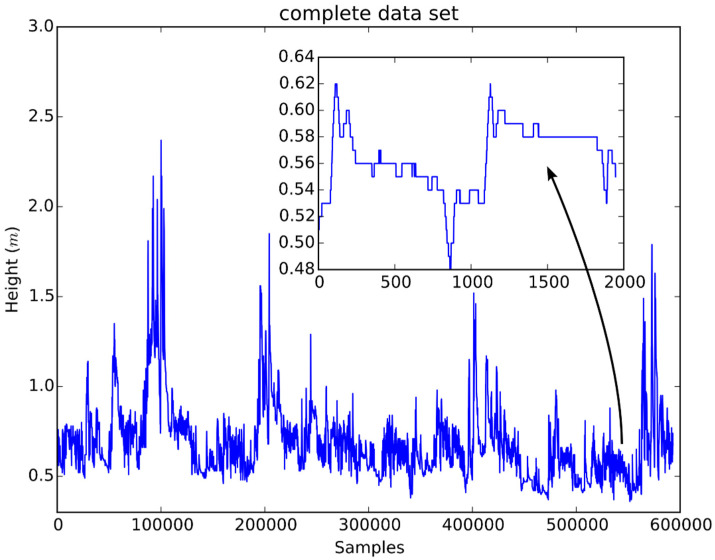
A plot of the entire data set of water level. The smaller plot embedded in the overall data plot shows a detail revealing small variations not visible otherwise.

The complete 10-min data base contained 602,928 readings from 2 February 1999 to 20 July 2010. The dataset has been revised previously by an expert technician, who has removed the first part of the records, as these were not valid, since during that lapse of time the sensor was not providing reliable measurements. For this reason, the data employed in the experiments were acquired between the 10 April 1999 17:00 and the 20 July 2010 23:50, with a sampling rate of 10 min, totaling 593,178 points. Figure 3 shows a plot of the entire data set and a detail revealing the variation within approximately 14 days.

As seen in the embedded subplot, there are long periods with no variation in the level of water. This fact raises the concern of carefully selecting the set of delays to be employed in the pool of available model inputs.

On the one hand, it must be assured that the water level dynamics are included instead of a (near) constant input signal, and on the other hand, the resulting set of training vectors must not include many duplicated input-output patterns. Experimentally, it was verified that a regression vector with delays up to 14 days would include both faster and slower dynamics of the water level data and prevent regressors of constant value.

**Figure 4:**
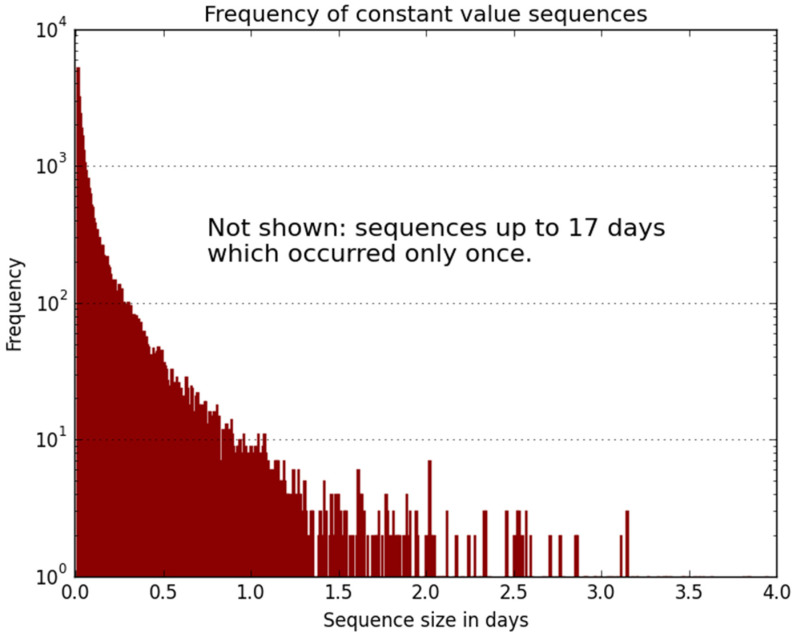
Frequency of constant and consecutive water level readings.

Figure 4 presents a histogram that clearly displays the frequency of constant consecutive water level reading sequences. It may be seen that a significant number of sequences have constant readings over more than one day and up to 17 days, although rare for more than two days.

## 3. Results and Discussion

Two MOGA model identification experiments were executed to identify a set of appropriate predictive water level models. Two main differences were considered in the second experiment concerning the model complexity and the search space.

### 3.1. First Experiment

The data set D was setup by constructing an input-output regressor matrix where each row corresponds to the regressor of a given time instant and each column corresponds to a certain output delay. The maximum delay considered corresponds to one week (1008), although the set from 0 to 1008 (recall the 10-min sampling interval) was decreased by using only subsets of delays extracted from various time intervals in the past:

- previous 12 h sampled every 10 min (as the original data);
- past 6 h sampled at every 20 min;
- past 6 h sampled at every 30 min;
- past 6 days sampled at every hour.

This resulted in a set of delays that, for a reference time instant k, includes more delays from the immediate past and fewer delays from 12 h in the past down to one week before k.

Due to the long sequences of consecutive constant water level measurements, illustrated in Figure 4. Four duplicated consecutive rows were removed from the data set matrix. This resulted in a D matrix having 570,507 regressor rows, each regressor having 246 delayed output values. The first half of D was used for training and generalization testing, while the second half was used for model validation.

The search space was defined by specifying the ranges for the numbers of neurons and inputs as detailed in Table 1. The inputs were selected from those pre-specified by the delays in the columns of matrix D.

The objectives were defined as the RMSE obtained in the training set D^t^, denoted by $\varepsilon^{t}$, and in the generalization testing data set D^g^, denoted by *ε^g^*. In the first case, the objective was set a restriction, $\varepsilon^{t}<0.0375$, and in the second case, it was set up for minimization. The MOGA parameters [24] were:

- population size: 100;
- proportion of random immigrants: 10%;
- selective pressure: 2;
- crossover rate: 0.7;
- survival rate: 0.5

As the RBF NN parameters were randomly initiated, 10 training trials were executed for each individual in the population to avoid poor parameter initialization. As the objective values need to be determined for each individual, the values concerning the trial that is closest (in the Euclidean sense) to the average are adopted. For each trial, 5% (14,262) of the first half of the regression matrix D were selected randomly. From these, 70% (9983) were used to train, while the remaining 4279 were employed for generalization testing. Each trial consisted of 50 iterations of the LM algorithm.

As no significant changes were observed in the objectives for a reasonable number of generations, the MOGA was stopped after 207 generations. Considering the minimized objective $\varepsilon^{g}$ and the restriction on $\varepsilon^{t}$, a non-dominated set of 16 models was obtained in the Pareto front. Table 2 presents a summary of the results obtained by the set of models where minimum, average and maximum values are shown. It should be noted that all values supplied to MOGA are scaled in the range [−1, 1].

**Figure 5:**
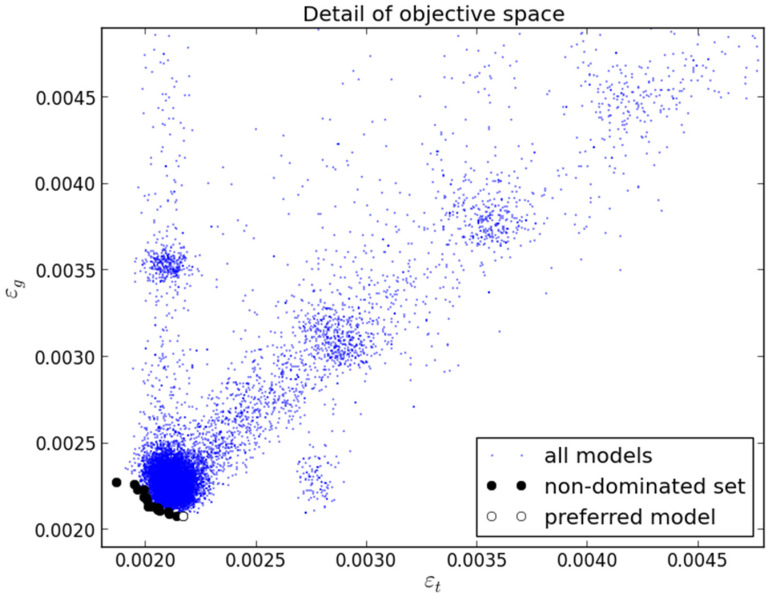
Detail of objectives obtained in the first experiment.

Figure 5 complements the results shown in Table 2 by showing a detail of a region of interest in objective space. It may be seen that the results converged in clusters of models to a larger aggregate dominated by the Pareto front. There are clusters that are better in one objective and several clusters that show a good compromise between both.

As only one objective was setup for minimization, the application of the preferability criterion [26] (Fonseca and Fleming, 1998) results in only one preferable model. It is marked as preferred in the figure and attained the lowest value on the generalization testing error. 

**Figure 6:**
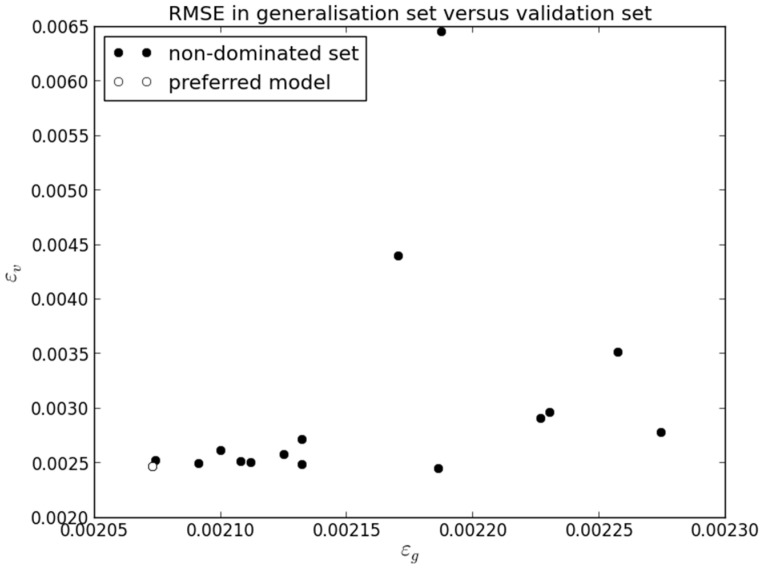
Validation results achieved by the 16 models obtained in the first experiment.

The 16 models were tested on the complete validation set (half of D: 285,254 points). The results are presented in Figure 6.

It may be observed that most models achieve excellent RMSE in the validation data set, independently of the result obtained in the generalization testing set. As in objective space, the preferred model achieves an excellent result (second best) in the validation data set. Collectively the results show that these models generalize very well to new data.

**Figure 7:**
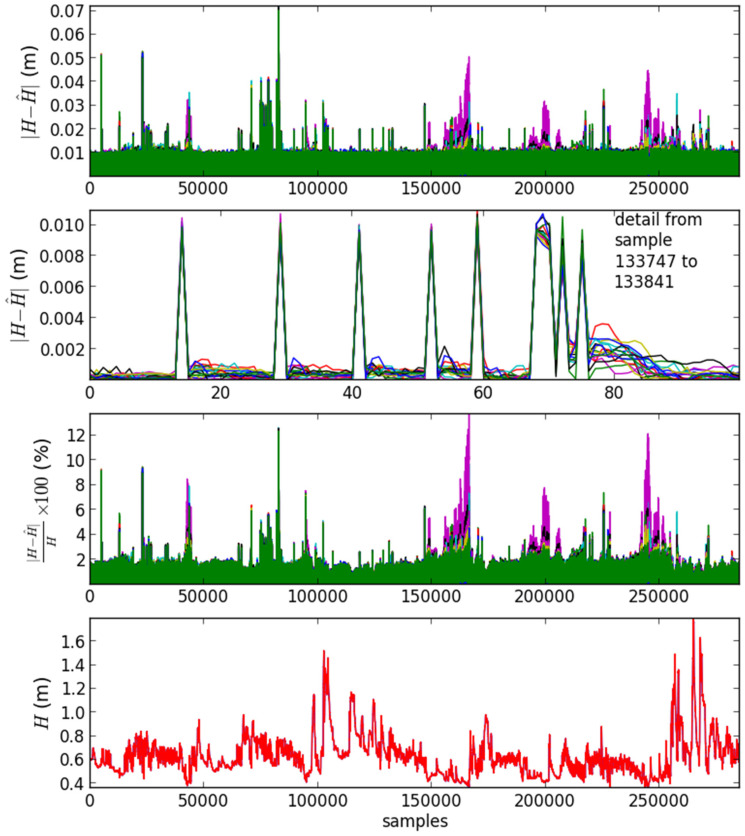
Graphical overview of the results obtained in the validation data set of the first experiment.

A graphical overview of the results in the validation data set is presented in Figure 7. Each different line color corresponds to one of the 16 models obtained. The plot at the top shows the absolute water level (*H*) error value, highlighting the small amplitude of error obtained. The second plot from top shows a detail of about 100 points showing the baseline error at around 0.001, with peaks one order of magnitude above in instants where the signal changes. The third plot from the top shows the model absolute percent error. It demonstrates that the error around 0.01 in the topmost plot corresponds to approximately 2% of the output (correspondingly, the 0.001 baseline corresponds to 0.2%). Finally, the plot at the bottom shows that the water level (H) model output is practically equal to the target.

Regarding model complexity, Table 3 presents the results obtained. Within the 16 models obtained, the number of inputs ranged from 29 to 46. For these input dimensions, the number of neurons varied from 11 to 24. Approximately half of the models employed 11, 12 or 13 neurons. These results correspond to models with 386 parameters (33 inputs, 11 neurons) up to 1105 parameters (44 inputs, 24 neurons).

### 3.2. Second Experiment

Although the results of the first experiment were excellent in terms of error performance and model generalization capacity, the models obtained were too complex, being large both in terms of the number of neurons as well as in terms of the number of inputs. Because of this, a second MOGA model identification experiment was executed with the aim of achieving the same predictive performance with less complex models. 

In the first experiment, the input terms selected in the 16 models, apart from the first delay present in all models, did not show high selectivity among the models. Therefore, in the second experiment, the pool of delays was decreased. As a result, the dimensionality of the regressor matrix was also decreased. As in the previous experiment, subsets of delays were extracted from various time intervals in the past:

- previous 4 h sampled every 10 min (as the original data);
- past 6 h sampled at every 20 min;
- past 8 h sampled at every 30 min;
- past 12 h sampled at every hour;
- past 18 h sampled at every two hours;
- past 5 days sampled at every six hours.

The regressors still range from a given instant down to that instant minus one week, but the pool of available delays decreased from 246 to 106. As before, consecutive duplicated regressors were removed from the matrix, resulting in a reduction from 570,507 to 523,421 points.

In terms of model structure search space, the same setup of experiment 1 was adopted (please see Table 1). Additionally, the MOGA parameters and the RBF training methodology were kept equal in the second experiment. Regarding the objectives, besides both RMSE objectives of experiment 1, an objective was included to minimize the number of parameters of the RBF models, given by,

(5) O=(n×(d+2))+1,

where n is the number of neurons and d is the number of inputs. The sum corresponds to the number of parameters of the Gaussian functions center positions (n×d) plus the spreads of Gaussian functions and the output linear combiner parameters (n×2+1).

The MOGA model identification framework was executed for 289 generations. At that point, the objectives had already converged, and no changes were observed in the Pareto front for a sufficient number of generations.

The execution resulted in a non-dominated set composed of 67 model instances, from which four were deemed preferable. Table 4 presents the results obtained in the objectives of the preferable set. The model complexity average value is deceiving, as the number of the parameters of models were 11, 13, 15 and 401.

**Figure 8:**
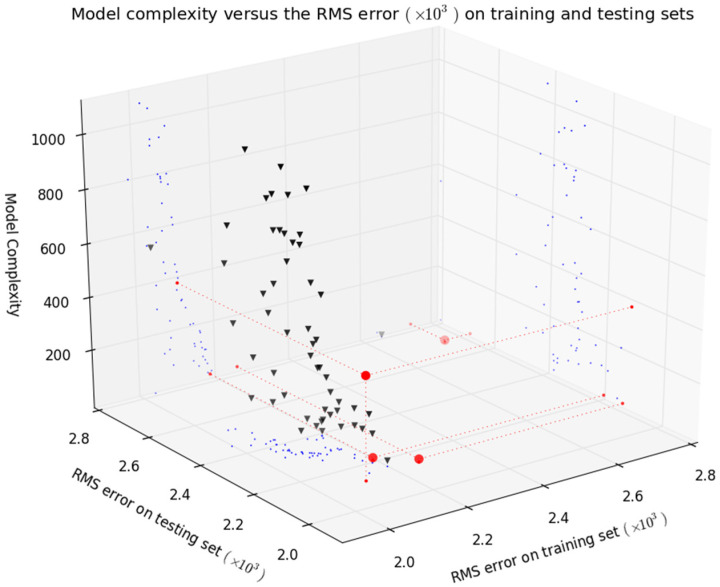
A detail of the objective values obtained in the second experiment. Triangles denote a non-dominated set and circles show results of the preferable set of models.

A detail of the results in objective space is shown in Figure 8. It may be seen that, except for one model, excellent accuracy results were obtained in the preferable set with low model complexity. Two of the four models achieve small RMSE values while having a small number of parameters. In this respect, the results are much better than those achieved in the first experiment. By comparing the RMSE values in Table 2 and Table 4, it may be seen that the huge reduction in model complexity (please recall values in Table 3) costed only a very small loss in predictive accuracy, well within the requirements of the model applications.

**Figure 9:**
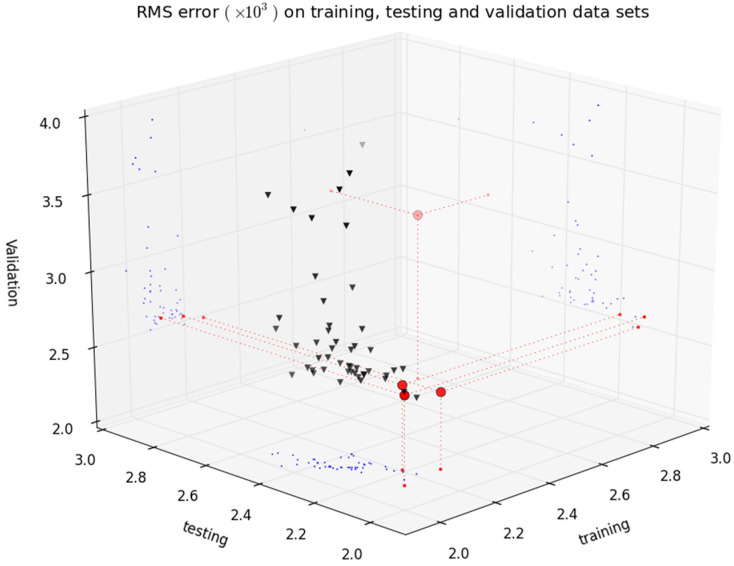
RMSE results in training, testing and validation sets obtained during the second experiment.

The four models in the preferable set were evaluated in the complete validation data set (261,711 points). The results presented in Figure 9 show that three of the four models achieve an excellent accuracy compromise between the three data sets. Importantly, the results of these three models are comparable (~0.0025) to those obtained in the first experiment (please see Figure 6). This means that the extra model complexity of experiment 1 contributed mainly to increase model accuracy in training and testing data sets, without affecting the accuracy on validation data.

The *z*-axis of Figure 8 presents the complexity of the models as defined in (5). Three models with only two neurons were achieved, corresponding to the three models that show a good compromise between RMSE in training, testing and validation data sets, highlighted in Figure 9. From these, considering the model complexity results, a model with five inputs achieved the best overall validation result.

Only 5 models in the 16 non-dominated models obtained in experiment 1 achieved marginally (by less than 0.00007) better validation results when compared to the selected model (highlighted by a different color in Table 5. If the input terms selected are translated to time delays, the model is

(6) $${\hat{H}}_{t+10\;min.}=f\left(H_{t},H_{t-10\;min.},\;H_{t-2h.\;\;50\;min.},\;H_{t-3h.\;\;20\;min.},\;H_{t-6h.\;\;20\;min.}\right),$$

where H is the water level and *J* is an RBF NN.

## 4. Conclusions

In this paper, it was shown how the MOGA framework can be applied to obtain a simple (in terms of complexity) model for 1-step-ahead prediction of water level in rivers. A comparison of the results obtained with other approaches is not straightforward, as different sampling times, different prediction horizons, different validation sets and different performance criteria are used in other alternative modelling techniques. 

As examples, the authors in [28] obtain an RMSE of around 1.3 × 10^−2^ for a 1-step ahead prediction in the validation set (with 591 samples), with a sampling interval of 1 h, for the river Tagliamento, in Italy. Our approach obtains an RMSE of 2.5 × 10^−3^ in a validation set with 261,711 samples, with a sampling interval of 10 min. In [29], the hybrid approach proposed obtains an RMSE of 5.6 × 10^−2^ for a 6-h prediction of the level of the Ouse River in England. The number of samples in the validation set is not specified. The authors in [30] use a wavelet-based artificial neural network and wavelet-based adaptive neuro-fuzzy inference systems for 1-setp-ahead prediction of daily water level of the Andog dam in South-Korea. The RMSE obtained for the years 2011-13 (around 26,280 samples) obtained ranged from 2.6 × 10^−2^ to 7.8 × 10^−2^. Noting that the previous results are not directly comparable to the results obtained in this paper, it can be broadly stated that the MOGA approach achieves RMSEs that are, typically, one order of magnitude smaller than the existing approaches. 

The present paper only addresses 1-step-ahead predictions. The existing models can be easily iterated to obtain predictions over a user-defined prediction horizon, as used in different applications of the same authors (see, for instance, [31,32,33]). This can be used for flood alarms.

Another application of these models is in the quality control of automated level stations using a data logger system similar to the one described in Figure 7 in [24] or using the model output as a fault-free data generator; sequential tests proposed in [12,34].

## Figures and Tables

**Table 1:** Model topology search space for the first experiment.

	Minimum	Maximum
Number of neurons	2	24
Number of inputs	2	48

**Table 2:** Summary of objectives obtained in the first experiment.

	$\varepsilon^{t}$	$\varepsilon^{g}$
Minimum	0.0019	0.0021
Average	0.0020	0.0022
Maximum	0.0022	0.0023

**Table 3:** Model complexity details of the models obtained in the first experiment.

**Number of Inputs:**	**29**	**31**	**32**	**33**	**34**	**35**	**36**	**38**	**39**	**44**	**46**
**Neurons:**	**1318**	**1321**	**1217**	**11**	**11**	**12**	**11**	**11**	**1123**	**24**	**1619**

**Table 4:** Summary of objectives obtained in the second experiment.

	$\varepsilon^{t}$	$\varepsilon^{g}$	O
Minimum	0.0021	0.0021	11
Average	0.0023	0.0023	110
Maximum	0.0027	0.0027	401

**Table 5:** Model complexity details of the preferred models obtained in the second experiment.

Number of Inputs:	3	4	5	38
Neurons:	2	2	2	10

## Data Availability

Not applicable.
